# Scrotal Abscess

**DOI:** 10.31662/jmaj.2021-0069

**Published:** 2021-09-01

**Authors:** Jun Kamei, Akira Kuriyama

**Affiliations:** 1 Emergency and Critical Care Center, Kurashiki Central Hospital, Kurashiki, Japan

**Keywords:** postsurgical, surgical site infection, perineum, abscess, processus vaginalis peritonei

A 92-year-old man underwent Hartmann’s colostomy formation and developed a surgical site infection. It was controlled with daily wound dressing, but a fascial breach remained. Hypotension with fever nonetheless persisted, and physical examination found the scrotum indurated (Figure 1). Ultrasonography and computed tomography showed a fluid collection within the scrotum (Figures 2 and 3), the drainage of which was purulent. The culture from the drainage isolated Extended-Spectrum Beta-Lactamase (ESBL) -producing *Escherichia coli* and *Enterococcus faecalis*. A diagnosis of scrotal abscess was established. Hypotension and fever resolved after drainage of the abscess.

Processus vaginalis peritonei can be patent at any age, leading to scrotal abscess, as typically seen in children with appendicitis ^(1), (2)^. In our case, bacteria isolated from the surgical site and scrotum were identical, implying that they might have migrated from the surgical site to his scrotum via the abdominal cavity and processus vaginalis peritonei. With this in mind, clinicians need to examine the perineum when the primary intra-abdominal infection is controlled but septic syndrome is persistent because processus vaginalis may be patent at any age.

**Figure 1.**
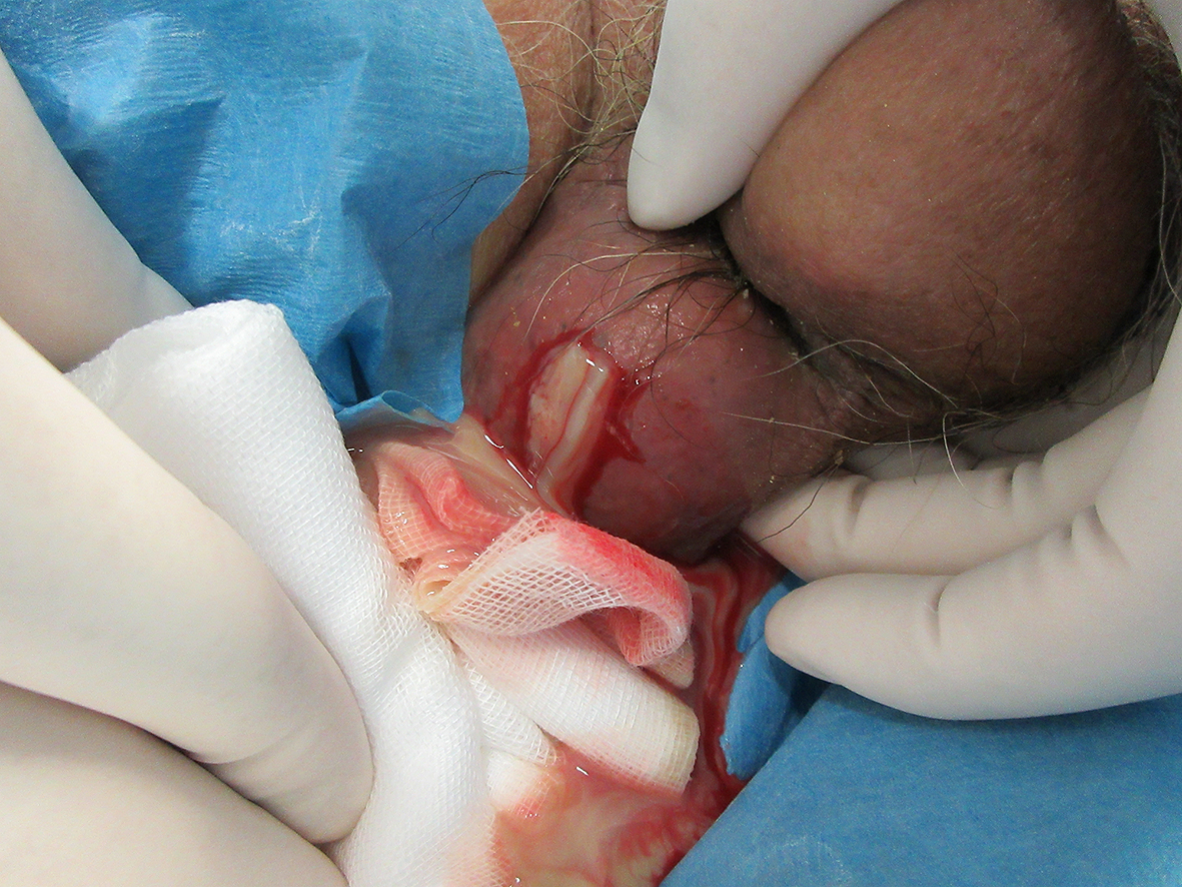
The scrotum was swollen with drained pus.

**Figure 2.**
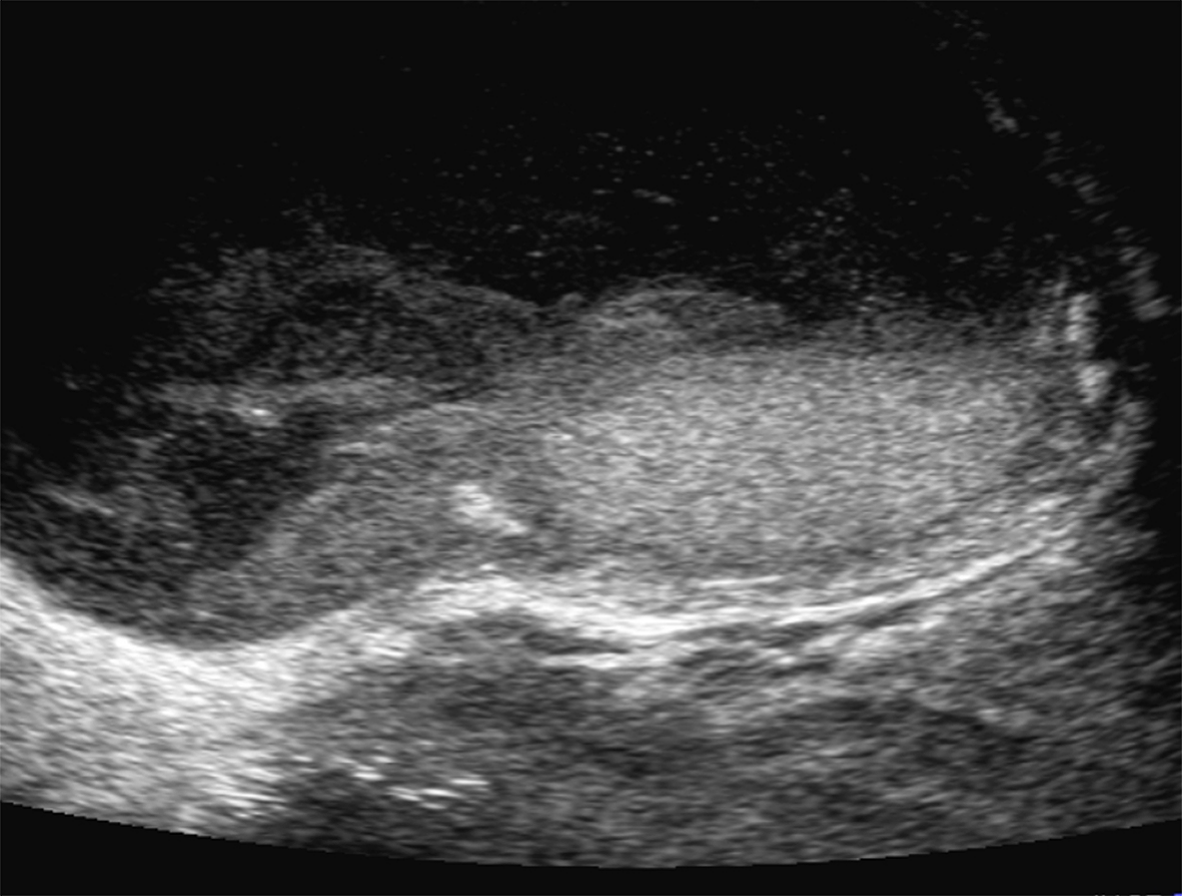
Ultrasonography showed scrotum with fluid collection.

**Figure 3.**
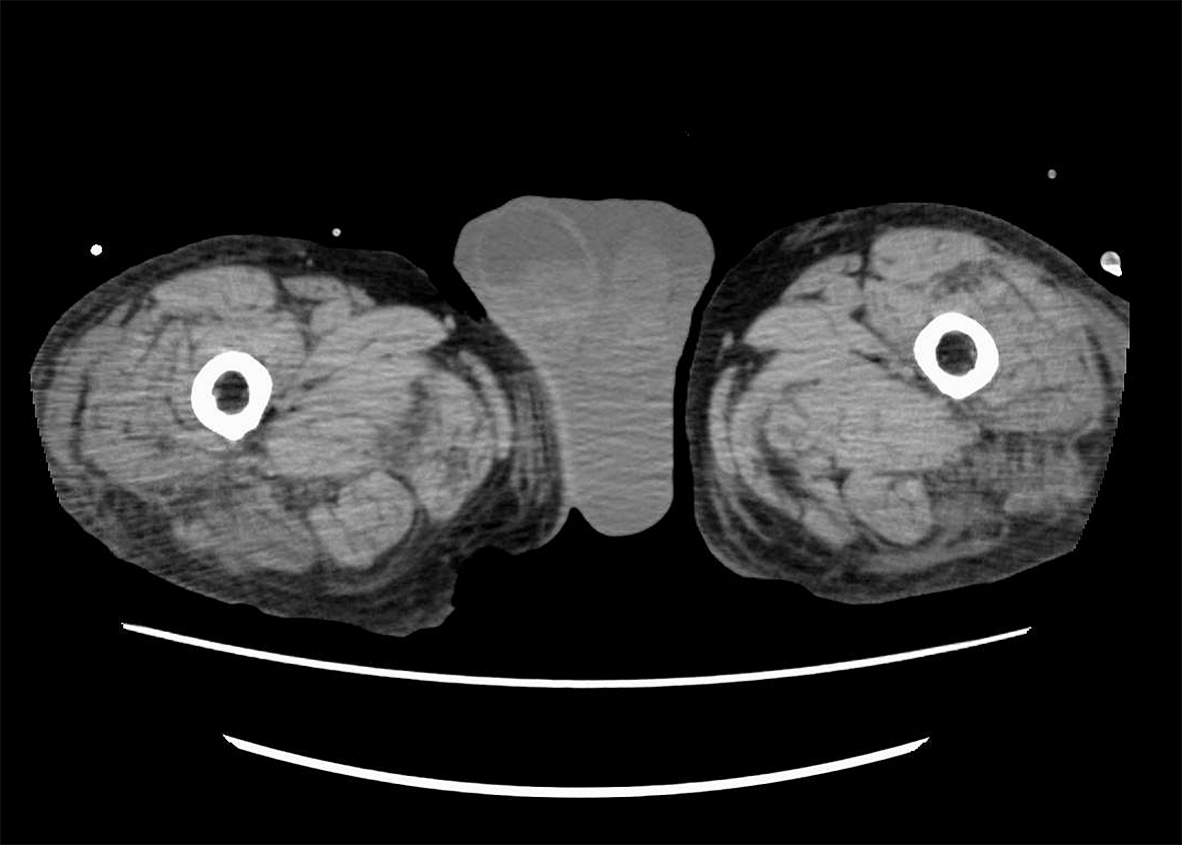
Computed tomography showed scrotum with fluid collection.

## Article Information

### Conflicts of Interest

None

### Author Contributions

JK wrote the manuscript. AK edited the manuscript.

### Approval by Institutional Review Board (IRB)

An IRB approval is not required because this is a case report.

### Informed Consent

Informed consent was obtained from the patient to publish the information, including photographs.
